# Social and Economic Impacts of COVID-19 Among Health Professionals in Vietnam: Status and Associations with Quality of Life and Sleep Quality

**DOI:** 10.1007/s44197-023-00156-7

**Published:** 2023-10-09

**Authors:** Linh Phuong Doan, Laurent Boyer, Pascal Auquier, Guillaume Fond, Bach Tran, Carl A. Latkin, Hien Thu Nguyen, Toan Van Ngo, Roger C. M. Ho, Cyrus S. H. Ho, Melvyn W. B. Zhang

**Affiliations:** 1 Institute for Global Health Innovations, Duy Tan University, Da Nang, 550000 Vietnam; 2 Faculty of Nursing, Faculty of Medicine, Duy Tan University, Da Nang, 550000 Vietnam; 3 Research Centre on Health Services and Quality of Life, Aix Marseille University, 27, boulevard Jean-Moulin, Marseille Cedex 05, France; 4 Bloomberg School of Public Health, Johns Hopkins University, Baltimore, MD USA; 5 Institute of Health Economics and Technology (iHEAT), Hanoi, Vietnam; 6 Department of Psychological Medicine, Yong Loo Lin School of Medicine, National University of Singapore, Singapore, Singapore; 7 Institute for Health Innovation and Technology (iHealthtech), National University of Singapore, Singapore, Singapore; 8 Lee Kong Chian School of Medicine, Nanyang Technological University Singapore, Singapore, Singapore

**Keywords:** Quality of life, Sleep quality, Healthcare worker, COVID-19, Vietnam

## Abstract

**Purpose:**

The COVID-19 pandemic has transformed the way of life of many individuals, especially those working at the frontlines, such as healthcare workers. Our study aims to examine the impact of COVID-19 on the socio-economic status, quality of life, and sleep quality when Vietnam was experiencing the 4th wave of the COVID-19 pandemic.

**Methods:**

A cross-sectional study was conducted on 604 healthcare workers using snowball sampling from October through to November 2021. Our study examined the impact of the government’s COVID-19 prevention policy including personal protective measures (5K measures), directive 15, directive 16, and directive 16 plus. The EQ-5D-5L and EQ-VAS were used to measure health-related quality of life and a scale of 1 to 10 was used to rate sleep quality of healthcare workers.

**Results:**

A total of 604 respondents, most people were female (57.9%), and working as civil servants (75.3%). Very few participants were able to increase their earnings during the pandemic. Participants who did not have monthly allowance amounts had the highest proportion (60.1%), followed by those under 2 million VND (21.2%). In the univariate regression model, people with high government policy scores tend to have lower quality of life and sleep quality scores. In addition, in the multivariable regression model, people with high scores on government policies tend to have lower quality of life (EQ-5D) scores.

**Conclusion:**

The COVID-19 prevention measures had a negative impact on quality of life, sleep quality, and daily demands of healthcare workers. These findings should help guide future policy implementations.

## Introduction

Vietnam first detected a case of COVID-19 on the 23rd, of January 2020 [1]. The Vietnamese government undertook rapid efforts to contain the spread of COVID-19, by not only forming a national steering committee, that oversaw and directs the coordinated actions of the government; but also implementing measures like lockdowns, in Son Loi [2]. To date, Vietnam has undergone four separate waves of COVID-19 infections. The first wave lasted between 23rd January to 16th April 2020 and was caused by the Wuhan virus strain from China [3]. The second wave of infection lasted between July 25th to December 1st, 2020 (caused by the D614G variant); 3rd wave lasted between January 28th to March 25th, 2021 (caused by the Alpha variant), and the 4th phase started from April 27th, 2021 onwards (caused by the Delta Variant) [3]. Since these waves, Vietnam has also experienced a recent resurgence of the pandemic caused by the Omicron variant, which was first detected in Hanoi on 19th January 2022 (4). To date, as of 26th May 2022, Vietnam has a total of 10,711,389 cases of COVID-19 infections, with a total of 43,076 deaths [4]. Some of the measures that helped in the rapid response and control of COVID-19 in Vietnam were that of contact tracing, isolation, and massive testing of individuals, as well as border control measures, such as border closures. In addition, the implementation of key social distancing measures has helped in curbing the spread of COVID-19 in the community [5]. Some of the key preventive measures are summarized in the table in Appendix 1, and include personal measures, as well as the implementation of several directives, such as Directive 15 and 16, and 16 plus. Measures such as Directive 15 state that all events that involve more than 20 participants were not allowed and members of the public have had to abide by a safe distancing of 2 m. By Directive 15, non-essential services were not allowed to go on. One of the other reasons that could account for the effectiveness of the governmental response was that the Vietnamese government has experienced having gone through other pandemics, such as the SARS-COV-1 pandemic and the H5N1 avian flu pandemics previously [5].

The COVID-19 pandemic has transformed the way of life of many individuals, especially those working at the frontlines, such as healthcare workers. A recent study undertaken by Pham et al. [6] have had examined the impacts of COVID-19 largely on the lifestyle and work of healthcare workers. The authors attempted to examine these impacts during the period when there was a nationwide partial lockdown (from April 7th to 14th 2020). The findings from the study revealed there are only marginal impacts of COVID-19 on healthcare workers. The fact that there remains limited impact has been attributed to the effective measures undertaken by the Vietnamese government. It was highlighted that the measures adopted by the Vietnamese government helped in ensuring that the healthcare system was not overly taxed and remained able in meeting the needs of individuals [6]. Other findings of interest were how participants felt there was a lack of appreciation by the public for the work they were doing, and the level of discrimination was dependent on the locality in which individuals worked at [6]. One of the limitations of the prior study was that it had not assessed the psychological impact of COVID-19 on healthcare professionals. In a recent study by Doan et al. [7], they looked at the psychological impact of COVID-19 on healthcare workers during the fourth wave of the pandemic. Of the 208 healthcare workers they sampled, they found that the rates of depressive symptoms, anxiety symptoms, and having a combination of depressive and anxiety symptoms were 38.94%, 25.48%, and 24.04% respectively. Predisposing factors towards these conditions include being in the younger age group, being single/widowed/divorced, or those who had to treat moderate to severe COVID-19 patients, and individuals who perceived themselves to be at a heightened risk of contracting COVID-19 at their workplaces.

Whilst these prior studies have provided insights into the impact of COVID-19 on healthcare workers, there remains to date no study that has explored the socio-economic impact, and how the pandemic has affected their health-related quality of life and sleep quality. The threat of COVID-19 on the economy has been previously articulated by Rasul et al. [8]. Their analysis reported that COVID-19 would not only affect a country’s economic growth rate but it might also result in fiscal deficit and increase the risk of macroeconomic instability. The threats due to the reduced income from travel and tourism would also affect small and medium businesses [8]. It is pertinent to study the impact of COVID-19 on the HRQOL (health-related quality of life), as COVID-19 might affect health status acutely, but the longer-term symptoms might have another consequential impact on health. Studies like that of Poudel et al. [9] have reported there is an impact of COVID-19 on HRQOL, and that individuals who were of female gender, older age, with more severe diseases at baseline, and patients from low-income countries were more susceptible.

Our study applied the Stress and Coping Theory and the Social Determinants of Health framework in our study to comprehensively investigate the impact of COVID-19 on healthcare workers' socio-economic status, Health-Related Quality of Life (HRQOL), sleep quality, and demands. The Stress and Coping Theory offers a well-established model for understanding individuals' responses to stressful events and the coping strategies they employ [10]. Notably, during the pandemic, healthcare workers have confronted unprecedented stressors, including fear of COVID-19 exposure, changing work conditions, and the risk of infection. Concurrently, the Social Determinants of Health framework is instrumental in contextualizing the experiences of healthcare workers, acknowledging the impact of individual characteristics and socio-economic factors on health outcomes [11]. Moreover, this framework facilitates exploration of potential disparities among healthcare workers arising from social and economic factors, aiding in the identification of vulnerable populations requiring additional support and resources [11]. By utilizing Social Determinants of Health Theory, we seek to gain valuable insights into the influence of stressors on the well-being of healthcare workers and the coping mechanisms they employ to navigate these challenges effectively. The integration of the Stress and Coping Theory with the Social Determinants of Health framework forms the foundation of our study's multi-dimensional analysis, enabling a comprehensive assessment of the impact of COVID-19 on healthcare workers' socio-economic status, HRQOL, and sleep quality. By combining the Stress and Coping Theory with the Social Determinants of Health framework, our study aims to provide a multi-dimensional analysis of the impact of COVID-19 on the socio-economic status of healthcare workers, and their HRQOL and sleep quality. The findings arising from this research are of importance in guiding new policy formulation, and in remediating any potential impacts. The conceptual framework employed in this study included predict variables (impact of COVID-19 on healthcare workers: individual characteristics, changing work, risk of COVID-19, and fear of COVID-19), mediating variables is personal protective measures; and outcome variables included HRQOL and sleep quality, as well as various demands. The findings arising from this research are of importance in guiding new policy formulation, and in remediating any potential impacts.

## Methods

### Study Design and Sampling Method

An online cross-sectional study was conducted from October through to November 2021. The conduct of this study was when Vietnam was experiencing the 4th wave of the COVID-19 pandemic. The snowball sampling method was utilized for the recruitment of participants. The snowball sampling method allows for the rapid collection of data, while also ensuring that the respondents fulfill the inclusion criteria. The questionnaire (as described below) was hosted on an online platform (https://surveymonkey.com), and the link to the questionnaire was disseminated to the respondents. This method was utilized as it helped ensure that data collation remains possible, in view of the COVID-19 restrictions, and allows for the rapid collation of responses.

The questionnaire that was used in this study was initially piloted among 15 staff members at the Vietnam Young Physicians Association. Their data was removed from the eventual analysis. The questionnaire link was then sent to a group of 20 respondents. Each respondent took approximately 30 min for the completion of the questionnaire. They were also told to assist in the invitation of more acquaintances and colleagues by sharing the link. The risk and benefits of participating in the study were explained to all respondents, and respondents were also aware that they were able to leave/withdraw from the study at any time.

With regard to data management, all the collated responses were stored on the SurveyMonkey web platform. The completion of the questionnaires and the progress of respondents were also monitored by means of the online site. The data was extracted from the site for further analysis.

### Participants

To be eligible for this study, participants needed to fulfill the following inclusion criteria: (a) Aged 20 years old and above; (b) Have been working in a medical facility in Vietnam for the last 6 months; (c) have had the means to assess the questionnaire. Participants were ineligible for the study if (a) they were not currently working or have not been working in a medical facility in Vietnam over the last 6 months; (b) have any underlying cognitive deficits that render them unable to complete the questionnaire; (c) do not have the means to access and complete the questionnaire.

There were 604 respondents who completed the questionnaire in its entirety.

### Questionnaires

The questionnaire used in this study included questions that asked about the following: (a) Demographic information of respondents; (b) Occupational information; (c) COVID-19 Vaccination status and perceived risk of exposure; (d) Fear of COVID-19; (e) Impact of COVID-19 on socioeconomic status; (f) Perception of the impact of government regulations and policies, (g) Health-related quality of life and sleep quality and (h) Changes in Consumer demands.

#### Demographic Information

The following questions were asked, which included: age group, gender, education, housing status, marital status, main monthly income, monthly household income, region, and area.

#### Occupational Information

The following questions were asked, which included: types of facilities currently working at (General hospital, Specialized/private hospital, CDC/Medical center/Public health station, University hospital, Other), Level of facilities (Central line, Province/City line, District line, Others), Specialty (Medical doctor, Nurse/midwife, Technician/administrative staff, Others), Contract status (Civil servant, Indefinite-term labor contract, Others), Working experience (Under 5 years, 5 years to under 10 years, Above 10 years, Under 5 years, 5 years to under 10 years, Above 10 years), Number of duties per week (None, 1 day, 2 days, 3 days and above), Working time (Under 8 h, 8 h, 9–10 h, 11–12 h, Above 12 h), Part time job (None, 1 2 and more).

#### COVID-19 Vaccination Status and Perceived Risk of Exposure

A question was asked to ascertain the number of vaccination doses that the participant has received. In determining the risks, participants were asked if they have had exposure to F0 (identified infected patients), exposure to F1 (Suspected people who have been in close/directed contact with patients), or contact with objects that may contain the virus (infected human utensils, patient samples). Participants were asked to rate their perceived risk of acquiring COVID-19 infection on a scale that ranged from non-risk to very high-risk. Similarly, participants were assessed for their parents, children, co-workers, and friends' perceived risk of contracting covid-19 on a scale ranging from no risk to very high risk.

#### Fear of COVID-19

Seven questions which were adapted from the Fear of COVID-19 scale (FCS-19), assessed how participants feel, think, or act toward COVID-19 [12]. The questions asked participants to rate how strongly they agreed with each statement (1: “strongly disagree”, 2: “disagree”, 3: “neutral”, 4: “agree” 5: “strongly agree”). The total score is calculated by the total score of 7 questions (range between 7 to 35 points) with the higher score indicative of greater fear of COVID-19.

#### Impact of COVID-19 on Socioeconomic Status

Questions were asked to determine if there was a change in earnings during the COVID-19 pandemic, and this was rated as “Decrease above 50%, decrease from 30%-50%, decrease under 30%, unchanged, increase under 30%, increase from 30 to 50%, increase above 50%”. We also asked about the amount of monthly allowance. In addition, the reasons for the increase and decrease in household income and expenditure were also incorporated into the questionnaire. The reason for extra income included: Increase in base salary, Bonus from the agency, Increase from pandemic prevention subsidy, No increase; The reason for the decrease in income included: Due to decrease in agency revenue, Due to end of project/sponsor/no job, No decrease, Others; Reasons to increase spending include: Spending on food, Medical expenses, Buy more items and supplies, No increase in spending, Reasons to reduce spending include: Someone lost their job, Save, reduce use. Finally, the ability to pay for living and the amount of money borrowed in the past year was also asked.

#### Perception of the Impact of Governmental Policies

The impact of governmental policies was assessed for the following domains, that of food consumption, education, and healthcare. Participants rated the extent of the impact on a scale that ranged from 1 to 5 (1 = No influence, 2 = Little influence, 3 = Moderate influence, 4 = Much influence, 5 = Very influence). The average score of each measure (personal protective measures: 5K measures, Directive 15, Directive 16, Directive 16 plus) ranges from 3 to 15.

#### Health-Related Quality of Life

The EQ-5D-5L questionnaire was developed by EuroQol Group in 2009 and validated in Vietnam. It includes 5 dimensions: mobility, self-care, usual activities, pain/discomfort, and anxiety/depression [13, 14]. For each dimension, the possible answers were: no problems, slight problems, moderate problems, severe problems, and extreme problems. The EQ-5D index then was converted on a scale from 0.5115 to 1. The alpha coefficient of the questionnaire was 0.750. Moreover, EuroQoL Visual Analogue Scale (EQ-VAS) was designed to measure the participants’ self-assessed health with the value ranging from 0 (worst imaginable health) to 100 (best imaginable health).

#### Sleep Quality

The question was used to assess sleep quality with the following content: “Over the past 2 weeks, how would you rate the quality of your sleep?” With answers ranging from 0 to 10 (0 = Extremely Bad and 10 = Extremely Good).

#### The Change in Consumer Demand Compared to Before the Pandemic

Questions were asked regarding family's needs compared to the months before the pandemic 6 basic needs: Eating, Electricity, water, bills, Education, Travel, Clothes, and Healthcare. The change of needs was divided into levels: decrease above 50%, decrease from 30%, decrease under 30%, unchanged, increase under 30%, increase from 30 to 50%, and increase above 50%.

### Statistical Procedures

Statistical analysis was performed using Stata software (version 15). Categorical variables were presented as frequencies with percentages while continuous variables were presented as mean and standard deviation (SD). The Chi-squared and Kruskal; Wallis Tests were used to calculate the difference in three anti-epidemic periods (Not participating in anti-pandemic, participated in anti-Pandemic from 1 to 3 months, participated in anti-pandemic for more than 3 months) based on characteristics. The Listwise deletion method was used to clean up the data before further analysis.

In this study, we used both univariate regression models and multivariate regression models. Multivariate Tobit Regression models were carried out to examine the impact of COVID-19 prevention measures (compulsory using personal protection measures, directives 15, 16, and 16 plus) and related demographic characteristics, work factors, and psychosocial status of health professionals. The stepwise forward strategies were used to find the minimal models by using a p-value of 0.2 with this regression. Tobit Regression models and Ordered Logistic Regression models (univariate regression models and multivariate regression) were utilized to explore the relationship between the impact of COVID-19 prevention measures on Health-related to quality of life (EQ-5D-5L, EQ-VAS, and sleep quality. A significant level of p < 0.05 was used.

## Results

Table 1 provides an overview of the demographic characteristics of the respondents. 53.5% of the respondents were aged between 31 and 40 years old. Approximately two-thirds were living in the city area and 62.9% living in the Northern region. 60% were married. Approximately 60% of those sampled have had an average household income from 5 to 10 million VND per capita and 42.4% of them have a monthly income per capita from 5 to 10 million VND.

**Table 1:** Demographic characteristics of respondents

Characteristics	N	%
Total	604	100.0
Age group		
21–30	198	32.8
31–40	323	53.5
> 40	83	13.7
Gender		
Male	254	42.1
Female	349	57.9
Education level		
Intermediate/college	113	18.7
Graduated	253	41.9
Post-graduated	238	39.4
Area		
City	380	62.9
Town	112	18.5
Rural, mountainous	112	18.5
Region		
Northern	365	60.5
Central	151	25.0
Southern	88	14.5
Housing status		
Private house with parents	346	57.3
Private house without parents	142	23.5
Rented house or others	116	19.2
Marital status		
Single/divorced/widowed	126	20.9
Married	478	79.1
Main income/month		
Under 5 million VND	132	21.9
5–10 million VND	365	60.5
10 million VND or above	106	17.6
Monthly household income per capita		
Under 5 million VND	239	39.6
5–10 million VND	256	42.4
10 million VND or above	109	18

Table 2 provides an overview of the work characteristics and impact of COVID-19 on the work of health professionals. Most of the participants were working as civil servants (75.3%). 53.3% of the participants were medical doctors, and 23.8% were nurses/midwives. 75.3% of the participants were working as civil servants, and 13.9% were on an indefinite-term contract.

**Table 2:** Work characteristics and impact of COVID-19 on work of health professionals

Characteristics	N	%
Type of workplace		
General hospital	277	45.9
Specialized/private hospital	111	18.4
CDC/Medical center/Public health station	140	23.2
University hospital	44	7.3
Other	32	5.3
Level of workplace		
Central line	133	22
Province/city line	225	37.3
District line	176	29.1
Others	70	11.6
Speciality		
Medical doctor	322	53.3
Nurse/midwife	144	23.8
Technician/administrative staff	77	12.7
Others	61	10.1
Contract status		
Civil servant	455	75.3
Indefinite-term labor contract	84	13.9
Others	65	10.8
Work experience		
Under 5 years	153	25.3
5 years to under 10 years	220	36.4
Above 10 years	231	38.2
Working seniority		
Under 5 years	213	35.3
5 years to under 10 years	200	33.1
Above 10 years	191	31.6
Number of duty per week		
None	165	27.3
1 day	140	23.2
2 days	168	27.8
3 days and above	131	21.7
Working time		
Under 8 h	90	14.9
8 h	269	44.5
9–10 h	128	21.2
11–12 h	69	11.4
Above 12 h	48	7.9
Part-time job		
None	475	78.6
1	112	18.5
2 and more	17	2.8
Change in workload		
No increase	226	37.4
Less than 20% increase	148	24.5
Increase from 20 to 50%	147	24.3
Over 50% increase	83	13.7
Change in working time		
No increase	255	42.2
Less than 20% increase	167	27.6
Increase from 20 to 50%	121	20
Over 50% increase	61	10.1
The period of participating in the fight against the COVID-19 pandemic		
None	144	23.8
1–3 months	201	33.3
3–12 months	95	15.7
Above 12 months	164	27.2

Table 3 revealed that the vast majority (89.1%) of the participants have been vaccinated with 2 doses oparticipants have been vaccinated with 2 doses of the vaccine. Relating to the perceived risk of COVID-19 infection, 1 in 10 healthcare workers reported that they are not at risk. Participants rated their colleagues’ risk of contracting COVID-19 on average 3.5 out of 5. Significant differences were found between COVID-19 vaccination status, COVID-19 exposure, self-perceived risk of COVID-19, Fear of COVID-19, and the duration in which one was involved in the anti-pandemic efforts.

**Table 3:** Risks of COVID-19

Characteristics	Not participating in anti-pandemic		Participated in anti-Pandemic from 1 to 3 months		Participated in anti-pandemic for more than 3 months		Total		p value
	n	%	n	%	n	%	n	%	
COVID-19 vaccination									
Not yet vaccinated	10	6.9	3	1.5	3	1.2	16	2.6	< 0.001
Had been vaccinated 1 dose	20	13.9	18	9	12	4.6	50	8.3	
Had been vaccinated 2 doses	114	79.2	180	89.6	244	94.2	538	89.1	
COVID-19 exposure									
Exposure to F0 (identified infected patients)	9	6.2	96	47.8	88	34	193	32	< 0.001
Exposure to F1 (Suspected people who have been in close/directed contact with patients)	27	18.8	40	19.9	112	43.2	179	29.6	< 0.001
Contact with objects that may contain the virus (infected human utensils, patient samples)	27	18.8	55	27.4	107	41.3	189	31.3	< 0.001
Never been in contact (directly or indirectly) with someone with COVID-19	102	70.8	66	32.8	76	29.3	244	40.4	< 0.001
Self-perceived risk of COVID-19									
No risks	22	15.3	15	7.5	17	6.6	54	8.9	< 0.001
Low risks	56	38.9	40	19.9	52	20.1	148	24.5	
Moderate risks	47	32.6	54	26.9	89	34.4	190	31.5	
High to very high risks	19	13.2	92	45.8	101	39	212	35.1	
COVID-19 infection confirmed in relatives (yes)	8	5.6	34	16.9	45	17.4	87	14.4	0.002

	Mean	SD	Mean	SD	Mean	SD	Mean	SD	p value
Risk of COVID-19 (1–5)									
Risk of parents in COVID-19	2.3	1.1	2.4	1.2	2.6	1.1	2.4	1.1	0.016
Risk of children COVID-19	1.9	0.9	2.0	1.1	2.3	1.1	2.1	1.1	0.043
Risk of friends in COVID-19	2.6	0.8	2.8	1.0	2.8	0.9	2.8	1.0	0.098
Risk of colleagues in COVID-19	2.9	1.0	3.5	1.1	3.4	1.0	3.3	1.0	< 0.001
Fear of COVID-19 (7–35)	20.2	5.6	19.1	5.9	18.1	6.0	18.9	5.9	0.005

**Fig. 1:**
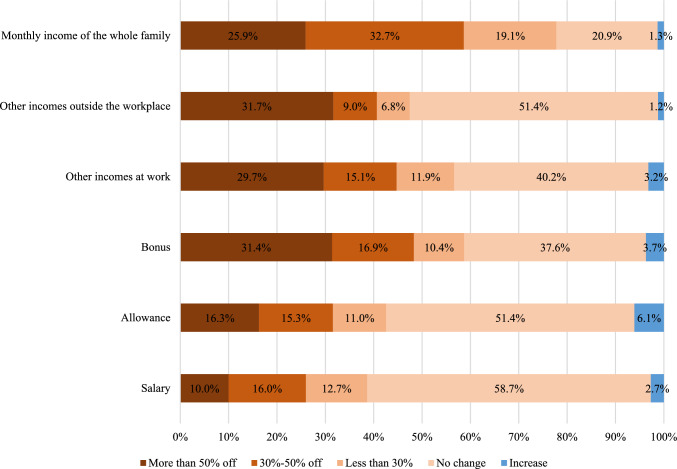
Change in earnings during the COVID-19 pandemic

Figure 1 illustrated the change in earnings during the COVID-19 pandemic. Very few participants were able to increase their earnings during the pandemic. Most of the household income of health workers has decreased (77.7%) while 38.7% of medical staff have had a pay cut. Just over 60% of health workers do not have allowances for COVID-19.

The impact of COVID-19 on socioeconomic status was presented in Table 4. Participants who did not have monthly allowance amounts had the highest proportion (60.1%), followed by under 2 million VND (21.2%), and more than 70% of respondents did not increase their income. A decrease in agency revenue was the main reason for the decrease in income (64.7%). The main cause of the increase in spending was spending on food (48.8%), by contrast, saving to reduce use was the main reason to reduce spending (64.6%). There were 63.4% of people partially sufficed for life and nearly 30% of participants borrowed over 50 million VND. The mean score of the EQ-5D index, EQ-VAS, and sleep quality was 0.87 (SD = 0.19), 79.4 (SD = 17.0), and 6.9 (SD = 2.0), respectively.

**Table 4:** Impact of COVID-19 on socioeconomic status

Characteristics	Not participating in anti-pandemic		Participated in anti-pandemic from 1 to 3 months		Participated in anti-pandemic for more than 3 months		Total		p value
	n	%	n	%	n	%	n	%	
Impact of the COVID-19 on economic status									
Monthly allowance amount									
Not available	118	81.9	100	49.8	145	56.0	363	60.1	< 0.001
Under 2 million VND	20	13.9	43	21.4	65	25.1	128	21.2	
2 million VND and above	6	4.2	58	28.9	49	18.9	113	18.7	
The reason for extra income									
Increase with base salary	15	10.6	25	12.4	41	15.9	81	13.5	0.285
Bonus from the agency	7	4.9	13	6.5	17	6.6	37	6.2	0.784
Increase from pandemic prevention subsidy	4	2.8	28	13.9	32	12.4	64	10.6	0.002
No increase	108	76.1	139	69.2	175	67.8	422	70.2	0.209
The reason for the decrease in income									
Due to decrease in agency revenue	87	60.4	128	64	175	67.6	390	64.7	0.344
Due to end of project/sponsor/no job	7	4.9	8	4	7	2.7	22	3.6	0.514
No decrease	39	27.1	43	21.5	50	19.3	132	21.9	0.192
Others	14	9.7	19	9.5	37	14.3	70	11.6	0.204
Reasons to increase spending									
Spending on food	65	45.1	102	50.7	128	49.4	295	48.8	0.572
Medical expenses	39	27.1	69	34.3	81	31.3	189	31.3	0.359
Buy more items and supplies	22	15.3	41	20.4	49	18.9	112	18.5	0.473
No increase in spending	58	40.3	69	34.3	95	36.7	222	36.8	0.528
Reasons to reduce spending									
Someone lost their job	39	27.1	43	21.4	50	19.3	132	21.9	0.191
Save, reduce use	87	60.4	128	63.7	175	67.6	390	64.6	0.337
Affordability for life									
Totally sufficed	19	13.2	38	18.9	50	19.3	107	17.7	0.399
Partially sufficed	101	70.1	123	61.2	159	61.4	383	63.4	
Totally not sufficed	24	16.7	40	19.9	50	19.3	114	18.9	
Loan amount in the past year									
Do not lend	64	44.4	71	35.3	94	36.3	229	37.9	0.639
Borrow less than 10 million VND	17	11.8	30	14.9	35	13.5	82	13.6	
Borrow from 10 to less than 50 million VND	25	17.4	42	20.9	48	18.5	115	19	
Borrowing over 50 million VND	38	26.4	58	28.9	82	31.7	178	29.5	

	Mean	SD	Mean	SD	Mean	SD	Mean	SD	p value
Impact of COVID-19 prevention measures									
Personal protective measures (5K measures) (3–15)	7.8	3.3	8.1	3.3	8.0	3.4	8.0	3.3	0.652
Directive 15 (3–15)	8.2	3.2	8.7	3.4	8.6	3.1	8.6	3.2	0.374
Directive 16 (3–15)	9.0	3.9	9.8	3.6	9.7	3.3	9.6	3.6	0.110
Directive 16 plus (3–15)	8.6	4.1	9.8	4.0	9.5	3.9	9.4	4.0	0.022
EQ-5D index (n = 1416)	0.88	0.15	0.87	0.20	0.86	0.20	0.87	0.19	0.509
EQ-VAS (0–100) (n = 1416)	83.1	14.1	80.7	16.6	76.4	18.3	79.4	17.0	0.001
Sleep quality (1–10)	7.5	1.7	6.9	2.0	6.6	2.0	6.9	2.0	< 0.001

**Fig. 2:**
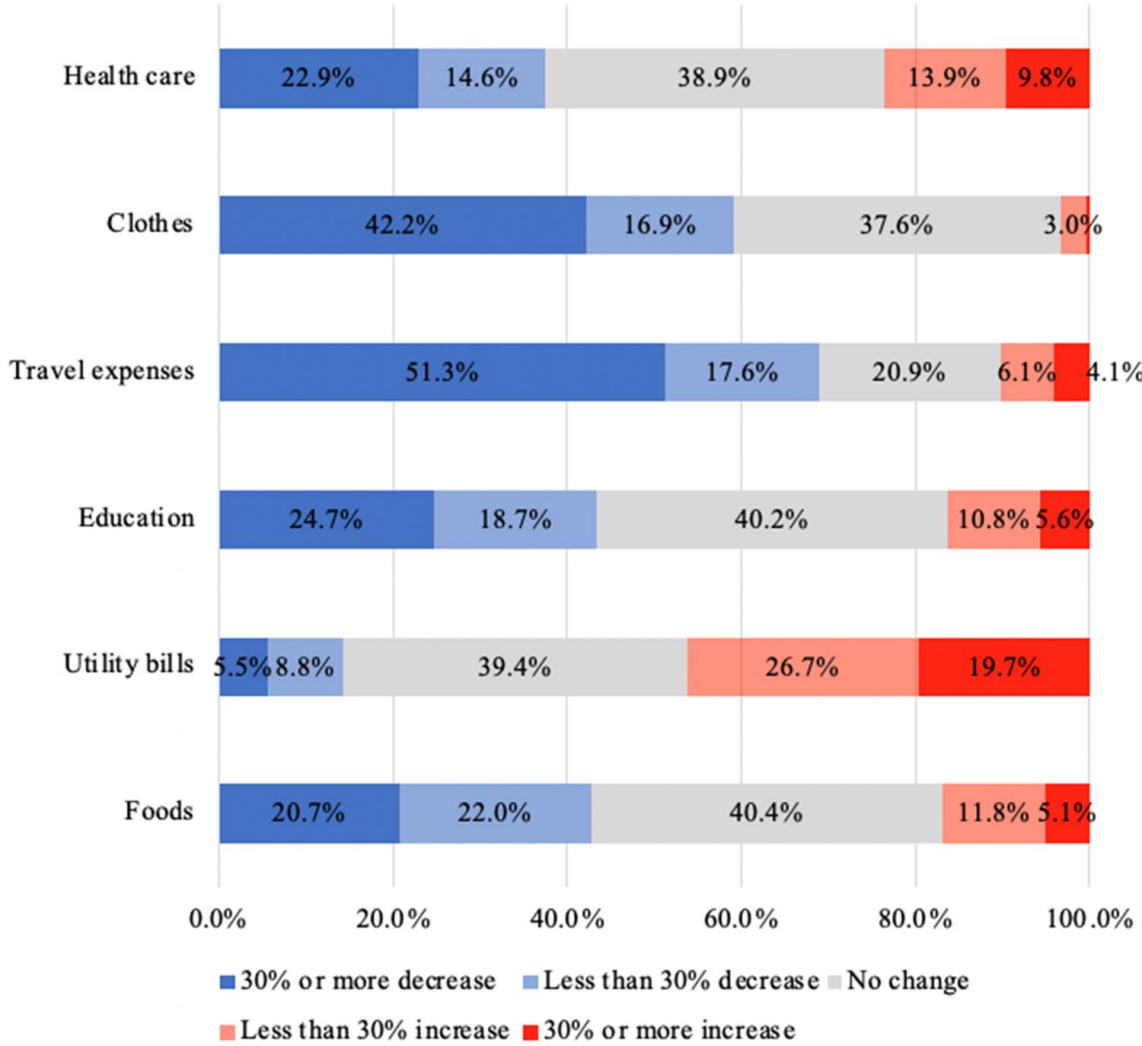
Changes in spending during the COVID-19 pandemic

Figure 2 presented that among the study participants, a high proportion of participants increased in spending for utility bills (46.4%), meanwhile, a high percentage of healthcare workers decreased in spending for travel expenses (68.9%), and clothes (59.1%).

Factors related to the impact of COVID-19 prevention measures among health workers are shown in Table 5. Graduates are more likely to be affected by government directives than those with high school/college education (Coef.: 1.26–2.47). Health workers with a main income of 5–10 million VND are less likely to be affected by Directive 16 than those with a primary income of less than 5 million VND (Coef.: − 1.55, 95% CI − 2.66; − 0.44). Compared with households with income below 5 million, those with income over 10 million tend to be affected by Directive 15, directive 16, and directive 16 Plus (Coef.: 1.16–2.73). Those who participated in the fight against the epidemic for less than 1 year were also more likely to be affected by the directives than those who did not fight the COVID-19 pandemic (Coef.: 1.22–1.87).

**Table 5:** Factors associated with the government's COVID-19 prevention policy

Variables	The impact of COVID-19 prevention measures (3–15)							
	Personal protective measures (5K measures)		Impact of directive 15		Impact of directive 16		Impact of directive 16 plus	
	Coef	95% CI	Coef	95% CI	Coef	95% CI	Coef	95% CI
Demographic characteristics								
Gender (vs male)								
Female			− 0.41	− 1.00; 0.18	− 0.67*	− 1.37; 0.04	− 0.64	− 1.54; 0.26
Gender (female vs male; ref.)								
Age group (vs 21–30)								
31–40	0.25	− 0.45; 0.94						
>40	1.28**	0.27; 2.30						
Education level (intermediate/college)								
Graduated	0.09	− 0.75; 0.93	0.74*	− 0.08; 1.57	1.59***	0.60; 2.57	0.79	− 0.44; 2.02
Post-graduated	0.70	− 0.18; 1.59	1.26***	0.39; 2.13	2.41***	1.37; 3.45	2.47***	1.15; 3.78
Housing status (private house with parents)								
Private house without parents							− 0.11	− 1.30; 1.08
Rented house or others							− 0.91	− 2.09; 0.28
Main income/month (vs under 5 million VND)								
5–10 million VND							− 1.55***	− 2.66; − 0.44
10 million VND or above							− 1.46*	− 3.05; 0.14
Monthly household income per capita (vs under 5 million VND)								
5–10 million VND	− 0.19	− 0.86; 0.47	0.25	− 0.38; 0.89	0.47	− 0.29; 1.23	1.20**	0.21; 2.20
10 million VND or above	0.82*	− 0.06; 1.69	1.16***	0.33; 1.98	1.27**	0.28; 2.26	2.73***	1.39; 4.08
Occuaptional characteristics								
Type of workplace (vs general hospital)								
Specialized/private hospital							− 0.11	− 1.30; 1.08
CDC/medical center/public health station							− 0.91	− 2.09; 0.28
University hospital							− 2.76***	− 4.51; − 1.01
Other							− 0.28	− 2.25; 1.70
Contract status (vs civil servant)								
Indefinite-term labor contract	0.01	− 0.87; 0.89	− 0.20	− 1.03; 0.63	− 0.31	− 1.31; 0.69		
Others	1.22**	0.18; 2.27	1.44***	0.47; 2.41	1.31**	0.13; 2.48		
Part time job (vs none)								
1	0.45	− 0.33; 1.22						
2 and more	− 1.53	− 3.43; 0.37						
The number of duty (vs none)								
1 day	0.88**	0.04; 1.73						
2 days	0.24	− 0.58; 1.07						
3 days and above	1.21***	0.31; 2.11						
The period of participating in the fight against the COVID-19 pandemic (vs none)								
1–3 months					1.22**	0.26; 2.17	1.57**	0.33; 2.80
3–12 months					1.61***	0.47; 2.75	1.87**	0.35; 3.38
Above 12 months					0.72	− 0.29; 1.73	0.96	− 0.41; 2.32
Economical impact								
Affordability for life (vs totally sufficed)								
Partially sufficed	− 0.13	− 0.98; 0.72						
Totally not sufficed	0.66	− 0.41; 1.74						
Increase income from pandemic prevention subsidy (yes vs no)	− 0.90	− 2.25; 0.45						
Reduced income because end of project/no job (yes vs no)	− 2.02	− 4.55; 0.51	− 2.92**	− 5.36; − 0.49	− 4.01***	− 6.89; − 1.13		
Increase spending because of buying food (yes vs no)							2.41	− 0.78; 5.60
Increase spending because of buying more items and supplies (yes vs no)	0.58*	− 0.08; 1.24						
Loan amount in the past year (vs do not lend)								
Borrow less than 10 million VND	0.15	− 0.82; 1.11	0.58	− 0.33; 1.50				
Borrow from 10 to less than 50 million VND	0.85*	− 0.02; 1.71	0.57	− 0.25; 1.39				
Borrowing over 50 million VND	0.84**	0.06; 1.61	0.85**	0.14; 1.56				
Risk of covid-19								
COVID-19 exposure (yes vs no)								
Exposure to F0			− 0.55	− 1.34; 0.24				
Exposure to F1					0.85**	0.08; 1.63		
Never been in contact with someone with COVID-19	− 0.80**	− 1.44; − 0.17	− 0.51	− 1.24; 0.21			− 1.32***	− 2.26; − 0.37
Self-perceived risk of COVID-19 (vs no risks)								
Low risks			− 0.04	− 1.17; 1.09	0.14	− 1.19; 1.47		
Moderate risks			0.14	− 0.97; 1.25	0.20	− 1.09; 1.50		
High to very high risks			1.05*	− 0.12; 2.22	1.25*	− 0.07; 2.57		
Fear of Covid-19 (range: 5–35, unit: score)	0.15***	0.09; 0.20	0.11***	0.06; 0.16	0.11***	0.05; 0.17	0.17***	0.10; 0.25

***p < 0.01, **p < 0.05, *p < 0.1

Table 6 gives information about the relationship between the impact of COVID-19 prevention measures on Health-related quality of life, sleep quality, and participant’s daily demands. In the univariate regression model, people with high government policy scores tend to have lower quality of life and sleep quality scores. In addition, in the multivariable regression model, people with high scores on government policies tend to have lower quality of life (EQ-5D) scores. People with higher government directives influence scores tend to have a higher need to pay other utility bills to have lower needs for clothing, transportation, and medical care.

**Table 6:** Impact of government policies on quality of life and demand for life among health professionals

Outcome variables	The impact of COVID-19 prevention measures (3–15)							
	Impact of personal protective measures (5K measures)		Impact of directive 15		Impact of directive 16		Impact of directive 16 plus	
	Coef (95% CI)	Coef (95% CI)^a^	Coef. (95% CI)	Coef. (95% CI)^a^	Coef. (95% CI)	Coef. (95% CI)^a^	Coef (95% CI)	Coef (95% CI)^a^
Tobit regression models								
Health-related quality of life and sleep quality								
EQ− 5D index (range: − 0.5115–1)	− 0.02*** (− 0.03; − 0.02)	− 0.02*** (− 0.03; − 0.01)	− 0.02*** (− 0.03; − 0.01)	− 0.02*** (− 0.02; − 0.01)	− 0.02*** (− 0.02; − 0.01)	− 0.01*** (− 0.02; − 0.01)	− 0.01*** (− 0.02; − 0.01)	− 0.01*** (− 0.02; − 0.01)
EQ− VAS (range: 0–100)	− 0.77*** (− 1.25; − 0.29)	− 0.35 (− 0.82; 0.12)	− 0.69*** (− 1.18; − 0.19)	− 0.24 (− 0.72; 0.24)	− 0.42* (− 0.87; 0.03)	− 0.09 (− 0.53; 0.34)	− 0.43** (− 0.83; − 0.03)	− 0.26 (− 0.65; 0.14)
Sleep quality (range: 0–10)	− 0.07*** (− 0.13; − 0.02)	− 0.04 (− 0.09; 0.01)	− 0.09*** (− 0.15; − 0.04)	− 0.05* (− 0.10; 0.00)	− 0.08*** (− 0.13; − 0.03)	− 0.05** (− 0.10; − 0.00)	− 0.05** (− 0.09; − 0.01)	− 0.03 (− 0.07; 0.01)
Ordered logistic regression models								
Daily demands (from 30% decrease or more to 30% increase or more)								
Demand for food	− 0.04* (− 0.09; 0.00)	− 0.03 (− 0.08; 0.02)	− 0.02 (− 0.06; 0.03)	− 0.01 (− 0.06; 0.05)	0.01 (− 0.03; 0.05)	0.02 (− 0.03; 0.07)	− 0.01 (− 0.05; 0.03)	− 0.00 (− 0.04; 0.04)
Demand for electricity/water/invoice	0.05** (0.00; 0.09)	0.06** (0.01; 0.11)	0.07*** (0.02; 0.11)	0.08*** (0.03; 0.13)	0.04** (0.00; 0.08)	0.05** (0.00; 0.09)	0.03 (− 0.01; 0.07)	0.04** (0.00; 0.08)
Demand for education	− 0.05** (− 0.09; − 0.00)	− 0.04 (− 0.09; 0.01)	− 0.04* (− 0.09; 0.00)	− 0.04 (− 0.09; 0.02)	− 0.04* (− 0.08; 0.00)	− 0.03 (− 0.08; 0.01)	− 0.05*** (− 0.09; − 0.02)	− 0.05** (− 0.09; − 0.01)
Demand for travelling	− 0.04* (− 0.09; 0.00)	− 0.04 (− 0.09; 0.01)	− 0.03 (− 0.08; 0.02)	− 0.02 (− 0.08; 0.03)	− 0.05** (− 0.09; − 0.01)	− 0.03 (− 0.08; 0.01)	− 0.06*** (− 0.10; − 0.02)	− 0.06** (− 0.10; − 0.01)
Demand for clothes	− 0.03 (− 0.08; 0.01)	− 0.01 (− 0.06; 0.04)	− 0.04 (− 0.08; 0.01)	− 0.02 (− 0.07; 0.04)	− 0.05** (− 0.09; − 0.01)	− 0.03 (− 0.08; 0.02)	− 0.05*** (− 0.09; − 0.01)	− 0.05** (− 0.10; − 0.01)
Demand for health care	− 0.05** (− 0.10; − 0.01)	− 0.04* (− 0.09; 0.01)	− 0.07*** (− 0.12; − 0.03)	− 0.07*** (− 0.12; − 0.02)	− 0.06*** (− 0.10; − 0.02)	− 0.07*** (− 0.11; − 0.02)	− 0.07*** (− 0.10; − 0.03)	− 0.07*** (− 0.12; − 0.03)

^a^Coef (95% CI): Model is adjusted for demographic characteristics, occupational characteristics, risk of COVID-19, economical impact variables

***p < 0.01, **p < 0.05, *p < 0.1

## Discussion

This paper fulfills an existing gap in the research literature, as it explored the socio-economic impact and the impact of COVID-19 on HRQOL and sleep, amongst Vietnamese healthcare workers. Our sampled participants were mainly civil servants and the vast majority were doctors or nurses. Only a minority of healthcare workers reported that they perceived themselves to be not at risk of contracting COVID-19. Factors that affected one’s perception of risk included that of their vaccination status, their level of exposure, their fear of COVID-19, and the total duration in which they were involved in the anti-pandemic efforts. With regards to socio-economic impact, whilst there was a reduction in income for most individuals (70.2%), respondents also reduced their spending. We found that demographic variables, such as level of education, income, and duration of participation in COVID-19 efforts are associated with the impact of the various COVID-19 preventive measures. With regards to HRQOL and sleep quality, those with higher scores for the impact of governmental policies tend to have corresponding lower HRQOL scores.

Our findings revealed that participants perceived themselves to be at risk of exposure to COVID-19, and their perception of their risk levels was associated with several factors. It is obvious that one’s risk is dependent on their vaccination status, level of exposure, and the duration that they are at the frontlines. One pertinent factor we found was how one’s fear of COVID-19 could affect their overall perception of risk. This is congruent and in-line with the findings of prior research. Mohsin et al. sampled a total of 737 participants and reported that 73.5% and 15.7% of healthcare workers have had experienced moderate to severe degrees of fear and anxiety [15]. They reported that factors moderating the severity of fear included that of gender and specialty [15]. Troisi et al. (2021) have highlighted how the fear of COVID-19 might have a consequential impact on one’s psychological well-being and occupational efficiency [16]. They found that one’s fear was moderated by personality factors such as levels of neuroticism and attachment styles (thus, if one has a fearful attachment style, they are more likely to experience increased fear). Governmental measures, such as those set out in Appendix 1 might be one way to moderate and reduce fear amongst healthcare workers. However, there needs to be further investigation into factors that could help reduce fear, such as improving the safety of the work environment, enhancing the levels of personal protection, ensuring that there is adequate personal protection equipment, etc. The fact that individuals with certain personality traits could be more vulnerable would suggest there is a need in looking out for the well-being of these individuals.

This study has explored the impact of the Vietnamese’s government COVID-19 measures on healthcare workers, which is an aspect that has not been previously explored in any other studies. Whilst only lower-income healthcare workers were affected by the stricter pandemic regulations, for household income, the opposite finding was observed, in that those with higher income levels were more affected by the stricter regulations. One of the possible reasons for this might be that healthcare workers could have family members involved in other business entities, and the tougher regulations might have led to a disruption of their business activities, thus affecting overall income. Also, whilst individuals who took part in pandemic work reported that they were affected by governmental regulations, those who did not take part were unaffected. This might be due to the adaptations that individuals at the frontline have had to make, in response to the changes in the regulations.

This study highlights that there has been a reduction in the income of healthcare workers and that individuals have largely reduced their spending, and have been saving more. This finding is not unexpected, as Tran et al. (2020) in their exploration of the impact of COVID-19 on the economic well-being of individuals from the general population, have reported that as many as 66.9% of their sampled respondents reported there to be a reduction in the overall family income due to the pandemic [17]. As highlighted by Tran et al., one of the main reasons for the reduction was that Vietnamese businesses were unable to export to the Chinese Market, which accounted for many of the exports [17]. It has been widely reported in the media how overwhelmed healthcare workers are, and how they have been unpaid for their efforts [18]. In fact, there has even been an initiative during the COVID-19 pandemic for the recruitment of recovered individuals, to render support, and to relieve the burden on healthcare workers [19]. Given these findings, it might be timely for policymakers to reconsider the pay of healthcare workers at the frontlines, and to consider additional payments to support their work. There remains a need to expand the existing pool of healthcare workers; and for those who are recruited to contribute to the efforts, there needs to be adequate training. Our study revealed that more than 40% of participants work more than 8 h per day, more than 50% of participants increased workload and working time, raise important concerns about work productivity and performance among healthcare workers in the context of the post-COVID-19 situation in Vietnam. Working long hours without adequate rest can lead to fatigue, burnout, and reduced concentration, potentially compromising the quality of healthcare services provided by these workers. As the healthcare system in Vietnam continues to recover and adapt to the post-pandemic context, addressing these productivity and performance challenges is crucial for ensuring the delivery of effective and efficient healthcare services. As highlighted by Tunio et al., work productivity also significantly affected work performance and financial performance As highlighted by Tunio et al., work productivity also significantly affected work performance and financial performance [20].

We also found an association between governmental measures and HRQOL and sleep. Individuals who were most affected by the governmental regulations reported an overall lower quality of life score. However, the scores on the EQ-5D index and EQ-VAS (0.88 and 83.1) remain to be consistent with the scores on the same measures when the population was sampled prior to the onset of the current pandemic [18]. Thus, whilst certain individuals did have a lower quality of life in view of the regulations, there was no drastic overall decline. The different directives implemented did have had an impact on the overall HR-QOL score, which is not unexpected, given that most of the directives curtail one’s social interaction with others.

The main strength of this study is that we managed to examine the socio-economic impact of COVID on healthcare workers and examine if the governmental regulations have had any impact on their overall quality of life and sleep. We managed to make use of an online mechanism to capture the data from respondents, and the sample size was relatively sizeable. Despite these strengths, we are aware that the method we used might have not led to the sample being representative of the population. Most of the data has been based on self-reported information.

## Conclusions

This is, to our knowledge, the first study to have explored the impact of COVID-19 on healthcare workers, pertaining to their quality of life and socio-economic impacts. The COVID-19 prevention measures had a negative impact on Health-related quality of life, sleep quality, and daily demands of healthcare workers. Our study's comprehensive analysis of the impact of COVID-19 on healthcare workers' socio-economic status, HRQOL, sleep quality, and demands significantly contributes to the existing knowledge base. By combining Stress and Coping Theory and the Social Determinants of Health framework, our research offers nuanced insights into the factors influencing healthcare workers' experiences during the pandemic. We believe that our findings will serve as a foundation for evidence-based policies and interventions aimed at safeguarding the well-being, sleep quality, and demands of healthcare workers, ultimately bolstering the resilience of Vietnam's healthcare system in the post-pandemic era.

## Data Availability

The datasets used and/or analyzed during the current study are available from the corresponding author on reasonable request.

## Appendix 1. COVID-19 Prevention Measures of the Government During 4th Wave

**Table:**

	Personal protective measures (5K measures)	Directive 15	Directive 16	Directive 16 plus
No public gathering	Implement the 5K measures Face mask: wear fabric face masks in public and crowed places, wear medical masks at medical facilities and quarantine camps Distance: keep distance when making contact with others Disinfection: wash hands regularly with soap, hand sanitizer; Disinfect surfaces/items frequently touched; keep house clean and airy No gathering: no gathering in crowds Health declaration: when experiencing symptoms of fever, coughing, shortness of breath dial Ministry of Health’s hotline or local health authorities for further instructions on safe health examination	Cancellation of events with more than 20 people per room Ban on gatherings of more than ten people outside offices, schools, and hospitals	Under the social distancing rules, everyone is required to stay home as much as possible and only go out under absolutely necessary circumstances Ban on gatherings of more than 2 people outside offices, school, and hospitals	Similar to directive 16, but stricter Under the social distancing rules “Ai ở đâu ở yên đó”, everyone is required to stay home as much as possible and only go out under medical emergency The government organizes to bring essential necessities to each house or other forms of “shopping for households”
Safe distance between each other		Keep a safe distance of 2 m	Keep a safe distance of 2 m	Keep a safe distance of 2 m
Services providers		Suspension of non-essential businesses and services Essential services will be allowed to operate	Suspension of non-essential businesses and services Essential services will be allowed to operate but must strictly follow pandemic prevention measures	Suspension of non-essential businesses and services Essential services will be allowed to operate but must strictly follow pandemic prevention measures
Transport activities		Restrictions on traveling from coronavirus-hit localities to others Restrictions on transporting passengers from Hanoi and Ho Chi Minh city to other localities	Ban on traveling from coronavirus-hit localities to others Suspension of public passenger transport services	Ban on traveling from coronavirus-hit localities to others Suspension of public passenger transport services

## Abbreviations

HRQOL: Health-related quality of life
FCS-19: Fear of COVID-19 scale
EQ-VAS: EuroQoL Visual Analogue Scale
SD: Standard deviation
